# Simultaneous Determination of Three Furanocoumarins by UPLC/MS/MS: Application to Pharmacokinetic Study of *Angelica dahurica* Radix after Oral Administration to Normal and Experimental Colitis-Induced Rats

**DOI:** 10.3390/molecules22030416

**Published:** 2017-03-07

**Authors:** Youn-Hwan Hwang, Hye Jin Yang, Jin Yeul Ma

**Affiliations:** KM Application Center, Korea Institute of Oriental Medicine, Daegu 41062, Korea; hyhhwang@kiom.re.kr

**Keywords:** *Angelica dahurica* Radix, imperatorin, isoimperatorin, oxypeucedanin, pharmacokinetics, colitis

## Abstract

In traditional oriental medicine, *Angelica dahurica* Radix (ADR) is used in the treatment of gastrointestinal, respiratory, neuromuscular, and dermal disorders. We evaluated the pharmacokinetic profiles of oxypeucedanin, imperatorin, and isoimperatorin, major active ingredients of ADR, in normal and 2,4,6-trinitrobenzene sulfonic acid (TNBS)-induced colitis rats. A rapid, sensitive, and validated UPLC/MS/MS method was established for evaluating the pharmacokinetics of three furanocoumarins. After oral administration of ADR (0.5 and 1.0 g/kg), blood samples were collected periodically from the tail vein. In colitis rats, the time to reach the peak concentration (T_max_) of imperatorin and isoimperatorin was significantly delayed (*p* < 0.05). Lower peak plasma concentrations (C_max_) and longer mean residence times for all furanocoumarins were also observed (*p* < 0.05) compared with normal rats. There was no significant difference in the area under the plasma concentration–time curve or elimination half-lives. Thus, the delayed T_max_ and decreased C_max_, with no influence on the elimination half-life, could be colitis-related changes in the drug-absorption phase. Therefore, the prescription and use of ADR in colitis patients should receive more attention.

## 1. Introduction

*Angelica dahurica* Radix (ADR; ‘Baek-ji’ in Korean, ‘Bai-zhi’ in Chinese) is a common ingredient in traditional formulas in Korean and Chinese pharmacopeia, such as Gumiganghwal-tang and Oyaksungi-san. In traditional oriental medicine, ADR has been used as an anti-inflammatory herbal medicine to treat respiratory diseases (e.g., common cold, nasal congestion), dermal disorders (e.g., acne, ulcer, carbuncle), pain (e.g., headache, toothache, rheumatism), and intestinal disorders (e.g., diarrhea, dysentery, chronic ulcerative colitis) [1,2]. In modern pharmacological studies, ADR has also shown anti-microbial, anti-inflammatory, anti-asthma, anti-hypertensive, and anti-cancer properties [3]. In particular, ADR improved the swelling and atrophic spots of colonic mucosal membranes in long-term clinical observations of colitis patients [2]. More than 70 coumarins have been identified from ADR [4]. Of these, furanocoumarins, including imperatorin, isoimperatorin, and oxypeucedanin, are major active constituents of ADR [5]. Similar to ADR, they have many reported biological properties. Imperatorin and isoimperatorin have anti-convulsant, vasodilatory anti-hypertension, anti-inflammatory, analgesic, anti-spasmodic, and anti-cancer activities [6,7]. Oxypeucedanin has anti-inflammatory, antibacterial, anti-fungal, and anti-cancer activities [8,9]. Therefore, the pharmacokinetics of the three furanocoumarins in ADR should be evaluated in various disease states to provide more information that could explain their efficacy and to better understand the pharmacological basis of their actions.

Several analytical methods for pharmacokinetic studies of ADR in rats have been developed. A high-performance liquid chromatography with ultraviolet detection (HPLC-UV) method has been reported for the determination of byakangelicin and oxypeucedanin hydrate in rat plasma [10]. A gas chromatography–mass spectrometry (GC-MS) technique was developed for the determination of oxypeucedanin and imperatorin in rat plasma [11]. Recently, two ultra-performance liquid chromatography tandem mass spectroscopy (UPLC/MS/MS) methods, with lower limits of quantification (LLOQ) of 5 ng/mL, run times exceeding 20 min, or requirements of large plasma samples, were applied to study the pharmacokinetics of coumarins derived from herbal medicine in rats [4,12]. Nevertheless, pharmacokinetic studies still require the development of more sensitive, rapid, and specific analytical methods for the simultaneous determination of the target analytes.

Therefore, this study developed and validated a sensitive UPLC/MS/MS method for the determination of oxypeucedanin, imperatorin, and isoimperatorin in rat plasma. Structural and functional alterations in the gastrointestinal tract, such as lumen pH, mobility, diarrhea, and ulceration, may affect the pharmacokinetic profiles of active constituents administered orally [13]. Therefore, we also investigated whether their pharmacokinetic profiles were altered in rats with experimentally induced colitis, because herbal medicines are most often administered orally.

## 2. Results and Discussion

### 2.1. UPLC/MS/MS Analysis and Method Validation

#### 2.1.1. Identification, Selectivity and Linearity

**Figure 1:**
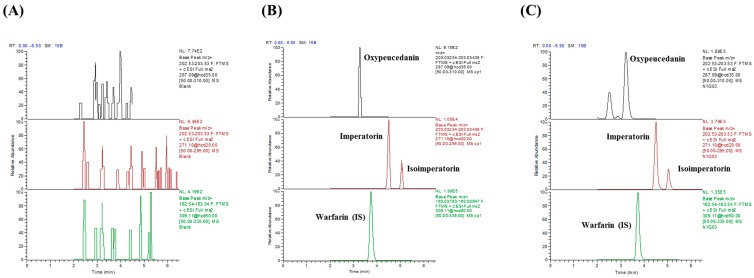
PRM chromatogram of the analytes and internal standard (IS) in plasma: (**A**) blank plasma; (**B**) spiked blank plasma in LLOQ; (**C**) plasma sample after oral administration of *Angelica dahurica* Radix.

**Figure 2:**
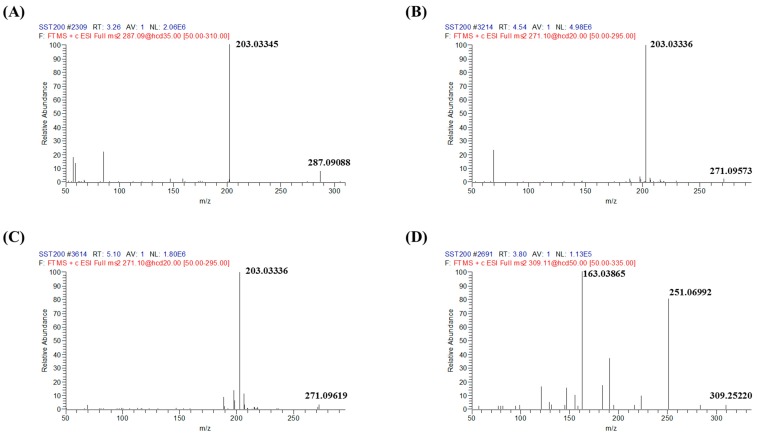
Mass spectrum of the analytes and internal standard (IS). Production *m*/*z* transitions for oxypeucedanin (**A**); imperatorin (**B**); isoimperatorin (**C**); and warfarin (IS, **D**) were selected 287.09088 → 203.03345, 271.09573 → 203.03336, and 309.25220 → 163.03865, respectively.

Under the established UPLC/MS/MS analytical conditions, there was no endogenous interference in the peak regions of oxypeucedanin (3.22 min), imperatorin (4.51 min), isoimperatorin (5.04 min), or an internal standard (IS, 3.74 min) in blank plasma with or without spiked analytes and authentic plasma samples (Figure 1). The most abundant and stable product ions of oxypeucedanin, imperatorin, isoimperatorin, and IS were *m*/*z* 203.03345 from 287.09088, 203.03336 from 271.09573, 203.03336 from 271.09573, and 163.03865 from 309.25220, respectively (Figure 2). Their calibration curves showed good linearity (r > 0.9994) and the lower limits of quantification (LLOQs) of all analytes with signal-to-noise ratios ≥20 were 1.0 ng/mL (Table 1).

#### 2.1.2. Accuracy, Precision, Recovery, Matrix Effects and Stability

The intra- and inter-day precision and accuracy were determined in analyses of QC samples for three concentrations of the three furanocoumarins, on the same day and on three different days. The intra- and inter-day accuracy and precision of all analytes was 6.9%–6.8% and 1.3%–9.4%, respectively (Table 2).

Table 3 lists the extraction recoveries, matrix effects, and stability of the analytes from rat plasma. The recoveries of the target analytes using liquid-liquid extraction ranged from 70.3% to 97.4%. Acetonitrile, used for sample preparation, showed good extraction efficiency, and the recoveries of all analytes at various concentrations were consistent and reproducible. The matrix effects ranged from 85.2% to 100%, and there was no obvious matrix effect. The stability of the target analytes was evaluated separately during the sample storing and processing procedures. The stability ranged from 94.4% to 101.6%, indicating that all target analytes in rat plasma were stable under our experimental conditions.

In this study, we focused on developing a simple, sensitive analytical method to apply to pharmacokinetic studies of oxypeucedanin, imperatorin, and isoimperatorin. Table 4 compares the analytical characteristics of our method with reported methods [4,11,12]. In pharmacokinetic studies, the volume of plasma/blood samples collected and continuous collection in the body can influence the pharmacokinetic parameters via a decrease in blood volume with blood loss. This proposed method uses less plasma and requires shorter run times compared with the reported methods [4,11]. The sensitivities of the target analytes were improved more than four-fold compared with those of the method reported by Chen et al. [12]. In summary, we developed a more sensitive, rapid, specific UPLC/MS/MS analysis, and a simple sample preparation method, for the simultaneous determination of three furanocoumarins for pharmacokinetic studies in rats.

### 2.2. Application of the Analytical Method in Pharmacokinetic Study

#### 2.2.1. Induction of Experimental Colitis

**Figure 3:**
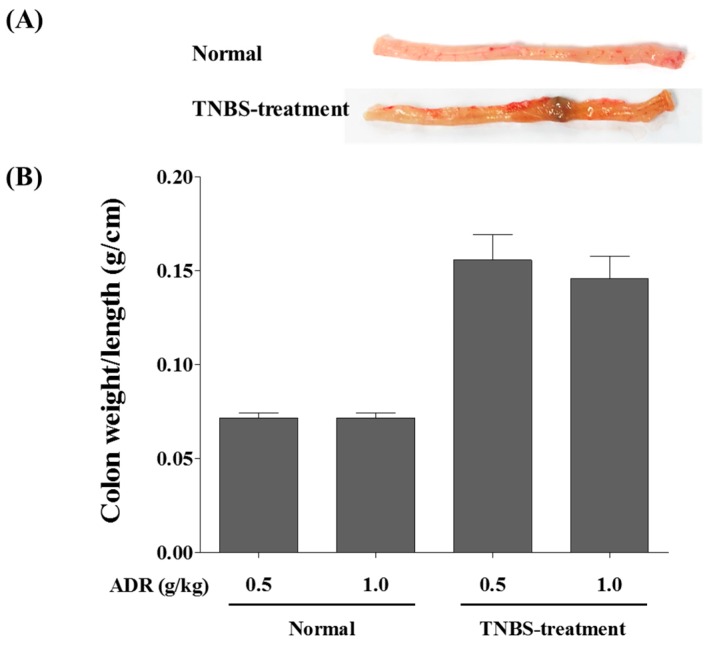
TNBS-induced experimental colitis in rats. (**A**) Macroscopic appearance of normal and damaged colon; (**B**) Weight of explanted colon.

In rats, the rectal administration of 2,4,6-trinitrobenzenesulfonic acid (TNBS) causes acute inflammation and severe damage to the intestinal barrier within three to seven days [14]. Ethanol disrupts the mucosal barrier and TNBS haptenizes autologous colonic and microbial proteins via the host immune system [15]. The induced-colitis rats in groups 3 and 4 developed bloody stools and lost 12.5% and 13.2% of their mean body weight, respectively. Necropsy showed that TNBS caused severe ulceration at the instillation site and a significant increase in colon weight (Figure 3). These observations are consistent with those of Arab et al. [16]. Therefore, the TNBS-treated rats can be used to evaluate the effect of colitis on the pharmacokinetics of the furanocoumarins in ADR.

#### 2.2.2. Pharmacokinetics of ADR in Normal and Colitis-Induced Rats

**Figure 4:**
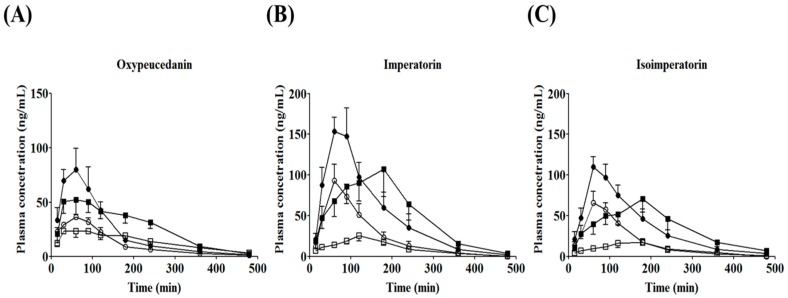
Plasma concentration–time curves of oxypeucedanin (**A**); imperatorin (**B**); and isoimperatorin (**C**) after oral administration of *Angelica dahurica* Radix (ADR, 0.5 and 1.0 g/kg, open and closed symbols, respectively) to normal (circle) and experimental colitis (square)-induced rats.

Figure 4 plots the mean plasma concentration–time curves of oxypeucedanin, imperatorin, and isoimperatorin after oral administration of ADR, and Table 5 summarizes their main pharmacokinetic parameters. The plasma concentrations of the three furanocoumarins in both the normal and TNBS-treated rats varied with the dose of ADR administered (0.5 or 1.0 g/kg). Our UPLC-MS/MS method was applied successfully to pharmacokinetic studies of oral oxypeucedanin, imperatorin, and isoimperatorin in normal and induced-colitis rats (*n* = 6), and the three furanocoumarins were confirmed to be bioavailable active components of ADR.

All three furanocoumarins reached peak plasma levels at 40–75 min in the normal rats, indicating rapid oral absorption. However, the times to reach the peak concentration (T_max_) of the three furanocoumarins were faster than in previous reports [11,12]. Zhao et al. [11] suggested that the pharmacokinetic profile of a single compound and the ADR extract after oral administration could differ as a result of the dosage form and composition. The doses of ADR used in this study were 4.5 and 9.0 times lower than those used by Chen et al. [12]. Excessive doses in pharmacokinetic studies could influence the pharmacokinetic parameters in the absorption phases [17]. In this regard, the difference in T_max_ might be due to differences in the amounts of furanocoumarin and non-furanocoumarin components as a result of the ADR preparation method and the dose of the test substance. In the colitis rats, the T_max_ of imperatorin and isoimperatorin was markedly delayed at 113–144 min (*p* < 0.05). After ADR administration, the peak plasma concentrations (C_max_) of oxypeucedanin, imperatorin, and isoimperatorin decreased significantly to approximately 50% (*p* < 0.05). Conversely, the mean residence times (MRT) of oxypeucedanin, imperatorin, and isoimperatorin were prolonged by 40%~65% (*p* < 0.05). The area under the plasma concentration–time curve (AUC_0→∞_) and the elimination half-lives (t_1/2_) of the three furanocoumarins were not significantly different between normal and colitis-induced rats. In colitis-induced rats, the delayed T_max_ and decreased C_max_ could be influenced in the drug absorption phase.

The changes in the physiological environment with gastrointestinal tract disorders, including colitis, can affect intestinal absorption with the oral administration of therapeutic agents, and can lead to therapeutic failure or adverse effects [18]. In colitis, functional disorders such as dyspepsia, intestinal hypomotility, and delayed gastric emptying are accompanied by structural changes resulting from inflammatory infiltrates, tissue swelling, and ulceration [19]. In particular, dyspeptic symptoms and gastroparesis, leading to impaired and delayed gastric emptying, occur frequently in colitis patients [20], and delayed gastric emptying can influence the pharmacokinetic parameters of oral drugs, such as T_max_ and C_max_ [21]. De Schepper et al. [22] reported that gastric emptying was delayed without a change in the upper intestinal transit via a neuronal pathway involving pelvic afferent nerve hyperactivity in TNBS-induced colitis rats. In addition, changes in CYP expression and the altered metabolic activity due to colitis could alter the plasma concentrations of drugs metabolized by CYP. Masubuchi et al. [23] found that the contents and activities of hepatic microsomes, such as CYP1A2, CYP2E1, CYP2C11, and CYP3A2, were decreased in TNBS-treated rats. Imperatorin and isoimperatorin are metabolized to xanthotoxol and heraclenin, respectively, via demethylation, hydrolysis, methylation, demethylation, and oxidation by liver microsomes, and these metabolites were identified in rat plasma, bile, and urine [24,25,26]. In the present study, TNBS-induced colitis resulted in a decrease in C_max_ and a delay in T_max_ without AUC_0→∞_, indicating impaired oral absorption of the three furanocoumarins. In this regard, further mechanistic studies of factors influencing their oral absorption, including under the above-mentioned conditions, are required in pathophysiological states.

## 3. Materials and Methods

### 3.1. Materials

**Figure 5:**
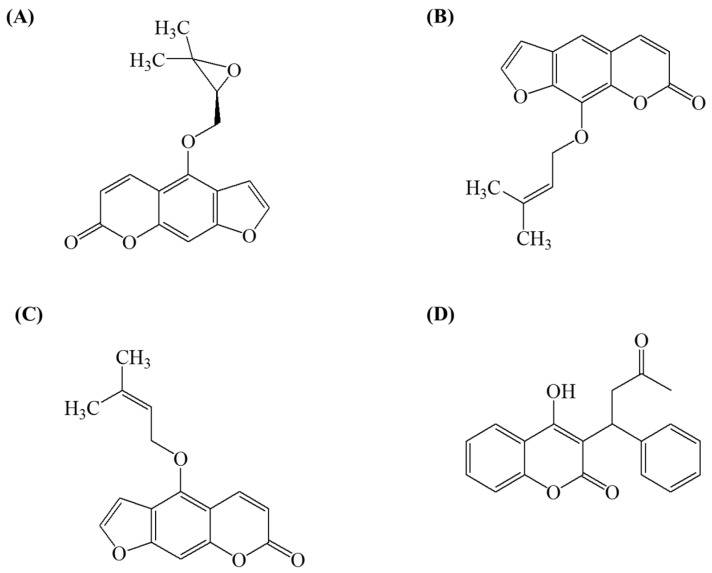
Chemical structures of oxypeucedanin (**A**); imperatorin (**B**); isoimperatorin (**C**); and warfarin (internal standard, **D**).

Imperatorin, isoimperatorin, and oxypeucedanin were purchased from ChemFaces (Wuhan, China; Figure 5). Warfarin as an IS, formic acid, ethanol, and TNBS were obtained from Sigma-Aldrich (St. Louis, MO, USA). “MS-grade” water and acetonitrile (Fischer Scientific, Loughborough, UK) were used for the MS analysis and plasma preparation. Raw ADR material was purchased from a local herb market (Yeongcheon, Korea) and stored in the herbarium (registration number #58) of the KM Application Center at the Korea Institute of Oriental Medicine. An ethanolic extract of ADR, a lyophilized brownish power, was prepared according to Jeong et al. [27]. The oxypeucedanin, imperatorin, and isoimperatorin contents in ADR were 6.67, 2.34, and 0.99 mg/g extract, respectively.

### 3.2. UPLC-MS/MS Analysis and Method Validation

Plasma concentrations of the three furanocoumarins were determined using an UltiMate 3000 (Dionex, Sunnyvale, CA, USA)—equipped Thermo Q-Exactive (Thermo Fisher Scientific, Bremen, Germany). A Hypersil GOLD column (50 mm × 2.1 mm, 1.9 μm, San Jose, CA, USA) and gradient method were used for chromatographic separation. A gradient elution (A, 0.1% formic acid in water; B, acetonitrile) with a 0.3 mL/min flow rate was programmed as follows: 35% B, 0–1 min, 35%–60% B, 1–3.5 min, and 60% B, 3.5–6 min. In the MS/MS analysis, positive ion mode and a parallel reaction monitoring (PRM) method were applied. The spray voltage (3.0 kV), capillary temperature (320 °C), sheath gas pressure (40 arbitrary units), auxiliary gas pressure (five arbitrary units), and resolution (17,500) were set to achieve optimal sensitivity and selectivity with the PRM method. Normalized collision energies for each analyte are shown in Table 6. To clean the plasma, each plasma sample (100 μL) was deproteinized with acetonitrile (200 μL) with IS (20.0 ng/mL), vortexed vigorously (5 min), and centrifuged (12,000 rpm, 10 min). Then, an aliquot of the filtered supernatant (0.22 μm, 3 μL) was injected into the UPLC/MS/MS system. Method validation was performed for linearity, selectivity, accuracy, precision, recovery, matrix effects, and stability, according to Zhou et al. [28]. Quality control (QC) samples (*n* = 6) at three concentration (2, 50, and 150 ng/mL) were used for method validation.

### 3.3. Animal Experiments

The animal study was approved by the Institutional Animal Care and Use Committee of Korea Institute of Oriental Medicine (Daejeon, Korea). Male Sprague-Dawley rats (245–260 g) were obtained from Samtako (Osan, Korea), housed under standard laboratory conditions with free access to food and water, and acclimated for seven days. Experimental colitis was induced using TNBS under isoflurane anesthesia, according to Arab et al. [16]. Before inducing colitis, the rats were fasted for 24 h. Under isoflurane anesthesia, the rats were given a single rectal dose of TNBS (50 mg/kg in 50% ethanol) through a medical grade polyurethane catheter (2 mm, external diameter) in the descending colon 8 cm from the anus sphincter. After slowly injecting the TNBS over 1 min, the rats were kept in a supine Trendelenburg position for 3 min to avoid intracolonic TNBS leakage. Normal rats received only 50% ethanol instead of TNBS, and the animals were used for the pharmacokinetic study on day 5 of the TNBS or 50% ethanol treatment. Before the pharmacokinetic study, no medication for pain control was administered, to avoid interrupting the colitis induction. Normal and induced-colitis rats were divided randomly into four groups (*n* = 6 each) (group 1, ADR 0.5 g/kg in normal rats; group 2, ADR 1.0 g/kg in normal rats; group 3, ADR 0.5 g/kg in induced-colitis rats; and group 4, ADR 1.0 g/kg in induced-colitis rats). ADR was prepared in distilled water, and administered orally by single gavage (5 mL/kg of dosing volume). Blood samples were collected periodically from the tail vein over 480 min without anesthesia. To obtain plasma, blood samples were centrifuged (12,000 rpm, 10 min), with the plasma stored at −80 °C before use.

### 3.4. Data Analysis

All values are presented as means ± standard deviation. A non-compartmental pharmacokinetic analysis for the time-concentration data of the three furanocoumarins was performed using the program PKSolver [29]. Comparisons of pharmacokinetic parameters between normal and TNBS-treated rats were performed using analysis of variance (ANOVA) or Student’s *t*-test with Prism 5.0. *p*-Values < 0.05 were considered to indicate statistical significance.

## 4. Conclusions

A rapid, sensitive UPLC/MS/MS method for the determination of three furanocoumarins was developed and used to evaluate the effects of colitis in a pharmacokinetic rat study. We first demonstrated that the colitis altered the rate and extent of oral absorption of major bioactive constituents after ADR administration, although the mechanisms are not clear. Thus, the prescription and use of ADR in colitis patients should receive more attention, and further studies are needed to clarify whether the altered pharmacokinetic profile is clinically relevant for therapeutic dosing.

## Figures and Tables

**Table 1:** Linearity and calibration curves of oxypeucedanin, imperatorin, and isoimperatorin.

Component	Range (ng/mL)	Linear Regression Equation	Correlation Coefficient	LLOQ (ng/mL)
Oxypeucedanin	1.0–200	*y* = 0.0444*x* − 0.0381	0.9996	1.0
Imperatorin	1.0–200	*y* = 0.0857*x* + 0.0136	0.9994	1.0
Isoimperatorin	1.0–200	*y* = 0.0431*x* − 0.0295	0.9995	1.0

**Table 2:** Accuracy and precision of oxypeucedanin, imperatorin, and isoimperatorin in rat plasma.

Components	Nominal Concentration (ng/mL)	Intra-Day (*n* = 6)			Inter-Day (*n* = 6)		
		Measured Concentration (ng/mL)	RE (%)	RSD (%)	Measured Concentration(ng/mL)	RE (%)	RSD (%)
Oxypeucedanin	2	2.1 ± 0.2	6.8	9.4	2.1 ± 0.2	6.8	8.1
	50	49.8 ± 1.7	−0.7	3.4	49.1 ± 2.6	−3.7	5.3
	150	152.4 ± 6.5	1.6	4.3	149.1 ± 1.9	−0.2	1.3
Imperatorin	2	1.9 ± 0.2	−6.9	8.3	1.9 ± 0.2	−4.9	8.7
	50	49.4 ± 1.9	−2.2	3.9	49.9 ± 2.3	−0.5	4.5
	150	149.1 ± 5.7	−0.6	3.8	151.7 ± 4.7	1.2	3.1
Isoimperatorin	2	1.9 ± 0.1	−5.1	7.0	2.0 ± 0.1	−4.1	4.4
	50	50.3 ± 1.8	1.0	3.6	48.6 ± 1.3	−5.6	2.8
	150	150.2 ± 3.0	0.1	1.9	151.4 ± 2.7	0.9	1.8

**Table 3:** Extract recovery, matrix effects, and stability of oxypeucedanin, imperatorin, and isoimperatorin in rat plasma.

Components/Nominal Concentration (ng/mL)	Recovery (%)	Matrix (%)	Stability (%)		
			Free-Thaw Cycles	At −70 °C for 30 Days	At Room Temperature for 24 h
Oxypeucedanin					
2	97.4 ± 7.9	100.2 ± 6.7	94.4 ± 6.4	97.7 ± 6.3	94.7 ± 7.6
50	84.6 ± 7.6	99.9 ± 2.2	99.9 ± 4.3	98.0 ± 0.8	97.8 ± 3.6
150	77.6 ± 7.4	100.8 ± 2.1	95.0 ± 8.8	99.6 ± 1.4	97.8 ± 6.6
Imperatorin					
2	84.9 ± 9.8	91.8 ± 7.6	97.4 ± 7.1	99.0 ± 6.4	97.6 ± 7.8
50	83.4 ± 3.7	92.3 ± 2.0	100.2 ± 3.3	101.0 ± 3.7	100.9 ± 5.0
150	79.8 ± 4.2	85.2 ± 3.1	101.1 ± 3.8	94.9 ± 9.7	101.6 ± 1.6
Isoimperatorin					
2	85.3 ± 12.2	94.9 ± 12.9	101.2 ± 4.6	98.3 ± 6.5	95.9 ± 7.4
50	75.6 ± 4.7	96.2 ± 6.9	98.8 ± 3.3	98.6 ± 3.6	100.3 ± 5.2
150	70.3 ± 7.6	94.6 ± 7.7	95.6 ± 9.2	97.1 ± 6.3	97.5 ± 7.2

**Table 4:** Comparison of the developed method and the previous method for determination of three furanocoumarins.

Methods	LC/MS/MS [4]	LC/MS/MS [12]	GC-MS [11]	UPLC-MS/MS (This Work)
LLOQ (ng/mL)				
Oxypeucedanin	1.16	5.00	7.00	1.00
Imperatorin	0.14	5.00		1.00
Isoimperatorin	0.14	4.00	10.00	1.00
Plasma sample amount (μL)	300	50	200	50
Run time (min)	27.0	5.5	50.0	6.0
Flow rate (mL/min)	1.0	0.3	1.2	0.3

**Table 5:** Pharmacokinetic parameters of oxypeucedanin, imperatorin, and isoimperatorin after oral administration of *Angelica dahurica* Radix to normal and experimental TNBS-treated rats.

Parameters	Normal		TNBS-Treated	
	0.5 g/kg	1.0 g/kg	0.5 g/kg	1.0 g/kg
**Oxypeucedanin**				
t_1/2_ (min)	78.1 ± 7.5	73.5 ± 7.8	91.9 ± 44.8	72.9 ± 7.7
T_max_ (min)	43.2 ± 11.2	49.1 ± 11.0	54.0 ± 22.8	42.4 ± 14.3
C_max_ (ng/mL)	38.5 ± 1.6	101.2 ± 21.2	29.0 ± 4.0 *	61.2 ± 11.9 *
AUC_0→∞_ (min·ng/mL)	5753.4 ± 649.8	10,866.7 ± 1720.6	7745.0 ± 1576.0	13,126.2 ± 2382.3
MRT (min)	125.9 ± 13.6	118.9 ± 11.0	203.7 ± 54.2 *	175.7 ± 7.8 *
**Imperatorin**				
t_1/2_ (min)	59.5 ± 7.2	61.5 ± 6.8	65.2 ± 11.5	56.1 ± 13.9
T_max_ (min)	54.0 ± 7.7	72.0 ± 6.7	113.7 ± 13.4 *	127.1 ± 18.4 *
C_max_ (ng/mL)	94.5 ± 19.7	201.4 ± 18.8	27.7 ± 6.3 *	118.5 ± 32.5 *
AUC_0→∞_ (min·ng/mL)	11,860.3 ± 2832.5	23,889.0 ± 4003.1	5196.0 ± 1349.7	24,845.0 ± 6485.8
MRT (min)	121.1 ± 10.4	127.6 ± 11.5	172.6 ± 12.8 *	187.6 ± 9.5 *
**Isoimperatorin**				
t_1/2_ (min)	63.8 ± 21.9	78.1 ± 2.4	88.0 ± 12.9	68.6 ± 24.2
T_max_ (min)	72.0 ± 6.7	67.5 ± 6.3	120.0 ± 16.7 *	144.0 ± 16.3 *
C_max_ (ng/mL)	72.1 ± 12.4	128.8 ± 8.7	21.1 ± 4.9 *	71.3 ± 16.2 *
AUC_0→∞_ (min·ng/mL)	9445.4 ± 2157.1	17,778.9 ± 2519.3	4197.9 ± 919.7	17,881.7 ± 4111.3
MRT (min)	143.2 ± 21.5	145.2 ± 11.6	204.3 ± 15.2 *	225.3 ± 33.5 *

t_1/2_, elimination half-life; T_max_, time to reach peak concentration; C_max_, peak plasma concentration; AUC_0→∞_, area under the plasma concentration–time curve from zero to infinity time; MRT, mean residence time. * *p* < 0.05, compared to normal rats treated with same dose.

**Table 6:** The information of PRM parameters.

Components	Chemical Formula	Precursor ion [M + H]^+^		Production	Normalize Collision Energy
		Calculated	Measured		
Oxypeucedanin	C_16_H_14_O_5_	287.09140	287.09088	203.03345	35
Imperatorin	C_16_H_14_O_4_	271.09649	271.09573	203.03336	20
Isoimperatorin	C_16_H_14_O_4_	271.09649	271.09619	203.03336	20
Wafarin (IS)	C_19_H_16_O_4_	309.11214	309.25220	251.06992	50
